# Special Issue “*Sporothrix* and Sporotrichosis”

**DOI:** 10.3390/jof4040116

**Published:** 2018-10-12

**Authors:** Héctor M. Mora-Montes

**Affiliations:** Departamento de Biología, División de Ciencias Naturales y Exactas, Campus Guanajuato, Universidad de Guanajuato, Noria Alta s/n, col. Noria Alta, C.P.; Guanajuato Gto. 36050, México; hmora@ugto.mx; Tel.: +52-473-732-0006 (ext. 8193)

**Keywords:** fungal pathogen, antifungal, immune system, diagnosis, *Sporothrix schenckii*, Feline sporotrichosis, *Sporothrix brasiliensis*, *Sporothrix globosa*

Sporotrichosis is a neglected, deep-seated fungal infection traditionally associated with *Sporothrix schenckii*, a dimorphic organism that was first described more than a century ago in human and rat specimens [1]. Since the disease caused by this organism is primarily confined to tropical areas, is not a nosocomial infection, or associated with high mortality rates, sporotrichosis is not regarded as a major threat to humans, like candidiasis, aspergillosis, and cryptococcosis [2]. However, the epidemiology, the clinical perspective, and fundamental aspects of *Sporothrix* have been changing significantly, and sporotrichosis is now considered a zoonotic disease, caused not only by one fungal species, and is associated with high morbidity rates, and, in the immunocompromised host, mortality caused by this infection is usual [3,4,5]. Thus far, there are several species that had been classified within the *Sporothrix* genus, but a handful of them, namely the pathogenic clade composed of *S. schenckii*, *Sporothrix brasiliensis*, *Sporothrix globosa*, and *Sporothrix luriei*, are related to sporotrichosis [6]. This Special Issue highlights the recent progress in the study of this disease and its etiological agents.

So far, *S. brasiliensis* has only been found in Brazil, whereas *S. globosa* predominantly in Asia; *S. schenckii*, on the other hand, is distributed worldwide [5]. As a confirmation of this trend, a study by Rojas et al. reports that *S. schenckii* is indeed the most prevalent species isolated from clinical specimens collected in Mexico, with the absence of *S. brasiliensis* and an anecdotic isolate of *S. globosa* [7].

Since the first description of the disease, its diagnosis has relied on the clinical manifestations, the direct examination of biological specimens, and the culturing of the microorganism [1]. However, the infection is pleiomorphic in terms of the clinical forms, which turns differential diagnosis into a difficult task, as revised here by Conceição-Silva and Morgado [8]. As proof of this, Tirado-Sánchez and Bonifaz revise the diagnosis of the sporotrichoid lymphocutaneous infection, the most frequent and characteristic form of sporotrichosis. They report a list of other fungal pathogens, bacteria, and viruses that can cause similar lesions in the skin, making differential diagnosis challenging [9]. As a helpful tool for clinicians, they propose an algorithm to facilitate the diagnosis of nodular lymphangitis caused by *Sporothix* spp. [9]. The microscopic examination of samples from lesions is not informative enough to establish a diagnosis of sporotrichosis, as the classical cigar-shape fungal cells or asteroid bodies found in cases of sporotrichosis are found in a small number of positive samples [10]. The culture of the fungus remains as the gold standard for the diagnosis of this infection, but it is time-consuming and culture-negative cases of sporotrichosis can occur. Here, Arenas et al. elegantly present a thorough revision of the literature, summarizing the new molecular and immunological alternatives developed to assist in the diagnosis of sporotrichosis [10]. Moreover, Bonifaz et al. evaluate the use of sporotrichin in skin testing for the diagnosis of cutaneous sporotrichosis [11]. The study, which included 138 cases of suspected sporotrichosis, demonstrates that the sporotrichin-based skin test showed a sensitivity and specificity for the disease of 94.5 and 95.2%, respectively, placing this assay as a complementary tool during the diagnosis of this disease [11].

The natural niche of *Sporothix* spp. has been traditionally related to vegetal tissues, debris, and decaying matter; this is the reason sporotrichosis is known as the rose pickers’ disease or the gardener’s disease [1,3,4]. Most recently, this infection has emerged as a zoonosis transmitted mainly by infected cats, which are causing severe epidemic outbreaks, like that reported in Brazil, caused by *S. brasiliensis* [12]. The current efforts to control the zoonotic transmission of sporotrichosis are not enough, as the number of cases in both humans and animals is not reducing [1]. One promising approach to break the infective cycle is to improve the diagnosis and treatment of ill cats, as the open lesions they have on their skin contain a high fungal burden [13]. Here, de Miranda et al. propose the evaluation of the fungal burden in open lesions as an indicator of the effectiveness of the antifungal treatment [13].

The specific environmental and ecological aspects of the natural habitat of the pathogenic species of the *Sporothrix* genus are poorly understood and limited information is currently available. Here, Ramírez-Soto et al. report a retrospective study aiming to establish the environmental conditions of the geographical locations where these organisms have been isolated from soil samples [14]. Sixteen countries are contained in the geographical regions where *Sporothrix* was commonly isolated, and *S. schenckii* was the most frequent species retrieved from these samples with an estimated temperature ranging from 6.6 °C to 28.84 °C and a relative humidity between 37.5% and 99.06% [14]. To provide more information about the environmental factors that influence the establishment of the infection caused by *Sporothrix* spp., Batista-Duharte et al. studied the effect of the exposure to mercury (II) chloride on the susceptibility to sporotrichosis [15]. This has a negative impact on the immunological fitness of mice, affecting the production of inflammatory cytokines, nitric oxide by macrophages, and lymphocyte populations [15]. The mercury-exposed mice showed higher fungal loads and propensity to develop the disseminated disease than the non-treated group, suggesting that sporotrichosis outbreaks could be related to polluted areas with a high content of mercury [15].

The antifungal repertoire to treat sporotrichosis usually depends on the clinical form of the disease, the longevity of the infection, the presence of other chronic illnesses in the patient, and the fungal species. Terbinafine, potassium iodine, and some azoles are among the drugs with good antifungal activity against *Sporothrix* spp., as discussed here by García-Carnero et al. [16]. Due to the limited options currently available to treat sporotrichosis and the constant menace of acquisition of antifungal drug resistance, the generation of passive and active immunological approaches has emerged as a new research area to control fungal infections, including sporotrichosis. The protein Gp70 has been one of the candidates to develop an anti-*Sporothrix* vaccine [17,18,19,20,21,22,23], but other alternatives, such as formulations using cell wall glycoproteins, have also provided promising results, which are underscored by the development of protective responses upon immunization with these preparations [24]. Here, Quinello et al., provide new information on the use of dendritic cells as alternatives to deliver fungal epitopes to stimulate a protective anti-*Sporothrix* response [25]. Mouse bone-marrow-derived dendritic cells were stimulated with *S. schenckii* cell wall proteins and then cocultured with splenocytes. As a result, the activated dendritic cells stimulated a cytokine profile suitable for the activation of the Th1 cell population, suggesting the cell wall preparation could lead to the establishment of a protective antifungal response [25]. Since the study was conducted in an in vitro setting, this observation still needs to be confirmed in the animal model of sporotrichosis.

Without doubt, the development of rational strategies to generate protective immunization against *Sporothrix* spp. or any other pathogen requires basic knowledge of the host–pathogen interaction, the main determinants that assist the invader in colonizing and damaging tissues, as well as the antigenic molecules this may possess and which are therefore recognized by elements of the immune response. Here, Conceição-Silva and Morgado and García-Carnero et al. summarize the current knowledge on the recognition of *Sporothrix* by the host immune system and identify the challenges that should be addressed in the near future [8,16].
